# Storage and stability of IgG and IgM monoclonal antibodies dried on filter paper and utility in *Neisseria meningitidis *serotyping by Dot-blot ELISA

**DOI:** 10.1186/1471-2334-8-30

**Published:** 2008-03-06

**Authors:** Aline S Ferraz, Elza FT Belo, Ligia MCC Coutinho, Ana P Oliveira, Andréia MS Carmo, Daniele L Franco, Tatiane Ferreira, André Y Yto, Marta SF Machado, Monica CG Scola, Elizabeth De Gaspari

**Affiliations:** 1 Immunology Section, Adolfo Lutz Institute, São Paulo, São Paulo, Brazil; 2 Bacterial Culture Collection, Adolfo Lutz Institute, São Paulo, São Paulo, Brazil

## Abstract

**Background:**

A simple filter paper method was developed for, the transport and storage of monoclonal antibodies (Mabs) at room temperature or -20°C after spotting on filter paper, for subsequent serotyping of outer membrane antigens of *N.meningitidis *by dot-blot ELISA.

**Methods:**

Monoclonal antibodies (Mabs) were spotted within a 0.5–1 cm diameter area of Whatman grade 903 paper, which were stored individually at room temperature or at -20°C. These MAbs were stored and analyzed after periods of one week, 4 weeks, 12 months, or 13 years in the case of frozen Mab aliquots, or after 4 weeks at -20°C or at room temperature (RT) in the case of Mabs dried on filter paper strips. Assays were performed in parallel using dot-blot ELISA. In addition to the MAbs specific for serotyping class 1, 2 or 3, we used a larger number of Mabs for polysaccharides, lipooligosaccharides (LOS), class 5 and cross-reactive antigens for native outer membrane of *N.meningitidis*. The Mabs dried on filter paper were eluted with phosphate-buffered saline (PBS) containing 0.2% gelatin.

**Results:**

Mabs of the isotypes IgG and IgM dried on filter papers were not affected by duration of storage. The detection by serotyping Mabs was generally consistent for dried filter paper MAb samples stored frozen for over 1 year at -20°C, and although decreased reactive antibody titers were found after storage, this did not interfere with the specificity of the Mabs used after 13 years as dry spots on filter paper.

**Conclusion:**

The use of filter paper is an inexpensive and convenient method for collecting, storing, and transporting Mab samples for serotyping studies. In addition, the samples occupy little space and can be readily transported without freezing. The efficiency of using immunoglobulin G (IgG) or M (IgM) eluted was found to be consistent with measurement of IgG or IgM titers in most corresponding, ascites Mabs stored frozen for over 1 year. The application of meningococcal typing methods and designations depend on the question being asked.

## Background

Meningococcal disease (MD) is a significant cause of mortality and morbidity throughout the world [1,2]. The incidence of MD in Brazil has been monitored since the occurrence of serogroup A and C epidemics between 1971 and 1974. In 1974, the incidence was greater than 179 cases per 100,000 inhabitants. From 1980 to 1992, the annual incidence of MD ranged from 1.0 to 1.4 per 100,000 inhabitants in different states of Brazil. During the period between 1981 and 1987, the mean proportion of serogroup B isolates identified was about 83%, while serogroup C strains represented only 6% of isolates. In 1988, the incidence of MD in the greater Sao Paulo area exceeded 4.06 per 100,000 inhabitants, suggesting a new epidemic in that region. This epidemic differed from previous ones because it was caused by serogroup B strains in 1988 and 1989 and serogroup B and C strains in 1990. The incidence of MD caused by *Neisseria meningitidis *serogroup C in greater São Paulo has been low since the end of the epidemic situation in 1971 and 1972. In that region, the prevalence of serogroup C strains increased from 4 to 14% and 8 to 32% during the years 1989 and 1990, respectively. Serotype 2b isolates were responsible for most of this increase, representing approximately 22 and 74% of the serogroup C strains isolated in 1989 and 1990, respectively [3,4]. In greater São Paulo, there has been a constant increase in the incidence of serogroup C meningococcal disease since the late 1980s [3,4].

The current serotyping system for meningococci is based on a battery of Mabs [5,6] which recognize antigenic differences in the outer membrane proteins of class 2 or 3 and 1, respectively [7]. The monoclonal antibody (Mab)-based typing system was developed because of the difficulties encountered with the use of absorbed hyperimmune polyclonal sera for typing.

After realizing the need for sensitive subtyping methods almost 20 years ago, an ambitious project to develop a Mab-based subtyping system was undertaken by researchers at The Netherlands National Institute of Public Health and Environmental Protection and by others. A panel of Mabs for serotyping and serosubtyping is now available at the website of (University of Oxford, UK). Before that dream was realized, an international interlaboratory comparisonof these reagents with 85 geographically and temporally diverse isolates of *N.meningitidis *serogroup B was was carried out in 1992 [8]. One of the problems with the Mab-based serotyping and subserotyping methods reported in that study was that a large proportion of isolates were nontypeable [8].

We described several years ago a simple method for the collection, preservation, shipment, and testing of minute amounts of dried monoclonal antibodies for typing *N. meningitidis *B [9]. The Mabs collected on filter paper were extracted with PBS and evaluated by dot-blot and immunoblot analysis employing whole cells of *N. meningitidis *B as antigen. The dried filter paper with Mabs could be stored at room temperature for as long as 30 days without detectable changes in antibody response when used for typing outer membrane antigens of *N. meningitidis *B in 1994 [9]. At that time, we used ascites and culture supernatant for two monoclonal antibodies of IgG isotypes specific for class 5 of *N meningitidi*s B by dot-ELISA and immunoblot, and subsequently, we performed a better characterization of the monoclonal [10]. We did not analyze Mabs of IgM and IgG isotypes for other outer membrane antigens of *N.meningitidis*, nor was there a study of long-term cold storage of filter papers with dried Mabs.

This paper describes a simple filter paper procedure for collecting monoclonal antibodies of IgG and IgM isotypes on Whatman grade 903 paper, which are then easily air-dried and stored at room temperature, or at -20°C for serotyping for *N.meningitidis*. The papers can then be easily transported from the clinic or field to the laboratory. A similar procedure has been widely used for collecting spots of dried blood or saliva to screen for various infectious agents [11-21], and metabolic and genetic diseases, for the presence of specific genes, also for forensic purposes. For several years, blood spot specimens on blotting paper have been frequently used for the diagnosis and seroepidemiologic investigation of infectious diseases. This method has been applied to the diagnosis and seroepidemiologic survey of bacterial, viral and parasitic diseases [22,23].

We also examined 135 *N. meningitidis *strains of serogroup A, 66 strains of serogroup C (from the 1972–1974 epidemics) from the culture collection at the Adolfo Lutz Institute, and 122 strains of serogroup B (from 1992) obtained from the reference center of *N. meningitidis *in Brazil. The National Reference Center for Meningitis (Adolfo Lutz Institute) serotypes all the Brazilian isolates of *N.meningitidis*. In our study, we also used reference strains for serogroups, serotypes, subtypes and immunotypes of *N.meningitidis *with a larger number of Mabs. The method described here permits the sending of Mabs to other Central Public Health Laboratory for serotying local isolates, thereby improving the epidemiologic monitoring of meningococcal infection.

## Methods

### Bacterial strains

We analyzed 135 *N. meningitidis *strains of serogroup A, 66 strains of serogroup C (from the 1972–1974 epidemics) from the culture collection at the Adolfo Lutz Institute, and 122 strains of serogroup B (from 1992) obtained from the reference center of *N. meningitidis *in Brazil, Adolfo Lutz Institute. In our study, we also used reference strains of *N meningitidis *in which cells were grown in (trypticase soy broth, Difco BRL products, Gaithersburg, MD) supplemented with 1% horse serum (Sigma, St.Louis, MO) in plates in a 5% CO_2 _atmosphere at 37°C [9]. The immunotype reference strains used were: 126E (L1), 35E (L2), 6275(L3), 89I(L4), 981(L5), M992 (L6),6155 (L7), M978 (L8), 120M (L9), 7880 (L10), 7889 (L11) and 7897 (L12).

### Monoclonal antibodies

The Mabs normally used for *N*.*meningitidis *typing belong to cell banks. Most of them are described at the website of (University of Oxford, UK). When new monoclonal antibodies are obtained, they must be compared with the reference strains [10] to determine whether they are new or have specificity comparable to that of existing antibodies. Tables 1, 2, 3, 4 describe the analysis of the Mabs studied here with the reference and case strains of *N.meningitidis*.

**Table 1:** Dot-blot ELISA using Monoclonal antibodies of serogroups for *N. meningitidis*.

				**1 week**		**4 weeks **		**1 year **		**13 years**	**4 weeks **	**RT**
Specificity	Mabs		isotypes	dilution(a)	dilution(b)	dilution(a)	dilution(b)	dilution(a)	dilution(b)	dilution(b)	dilution(a)	dilution(b)
Group A	14-1-A	WRAIR 2D7B5B5B2	IgG1	1:25,000	1:25,000	1:25,000	1:25,000	1:25,000	1:25,000	1:5,000	1:25,000	1:25,000
Group B	2-2-B	WRAIR 5C1-3H7	IgM	1.50,000	1.50,000	1.50,000	1.50,000	1.50,000	1.50,000	1:5,000	1:25,000	1:25,000
Group C	4-2-C	WRAIR 7H9-4	IgG3	1:25,000	1:25,000	1:25,000	1:25,000	1:25,000	1:25,000	1:5,000	1:25,000	1:25,000
Group W135	7-1-W	WRAIR 6G9-7	IgM	1:10,000	1:10,000	1:10,000	1:10,000	1:10,000	1:10,000	1:10,000	1:10,000	1:10,000
Group 29EB-1-29E	WRAIR	IgM	1:5,000	1:5,000	1:5,000	1:5,000	1:5,000	1:5,000	1:2,500	1:5,000	1:5,000	
Group Z	2C2-4.6	WRAIR	n/d	1:20,000	1:20,000	1:20,000	1:20,000	1:20,000	1:20,000	1:5,000	1:20,000	1:20,000
Group Y	5-2-Y	WRAIR 2C2-4.2-G6	n/d	1:2,500	1:2,500	1:2,500	1:2,500	1:2,500	1:2,500	1:2000	1:2,500	1:2,500

n/d = not done.

WRAIR = Walter Reed Army Institute of Research (USA).

RT = Room Temperature (30 days).

dilution(a) (Mabs) ascites freezer -20°C.

dilution(b) (Mabs) eluted of dried filter paper.

Mabs = monoclonal antibodies

**Table 2:** Dot-blot ELISA of serotypes and subtypes using Monoclonal antibodies for *N. meningitidis*.

**A**												
				**1 week**		**4 weeks**		**1 year**		**13 years**	**4 weeks**	**RT**
Specificity	Mabs		isotypes	dilution(a)	dilution(b)	dilution(a)	dilution(b)	dilution(a)	dilution(b)	dilution(b)	dilution(a)	dilution(b)
P2.2a	1-1-P2a	WRAIR 5D4-5	IgG3	1:100,000	1:100,000	1:100,000	1:100,000	1:100,000	1:100,000	1:5000	1:100,000	1:100,000
P2.2b	3-1-P2b	WRAIR 2H10-2	IgG3	1:5000	1:5000	1:5000	1:5000	1:5000	1:5000	1:500	1:5000	1:5000
P2c	5D9-5.6(5-1-P2c)	WRAIR	n/d	1:500.000	1:500.000	1:500.000	1:500.000	1:500.000	1:500.000	1:5000	1:500.000	1:500.000
P3.4	15-1-P4	WRAIR 5DC4C8G8	IgG2b	1:2000	1:2000	1:2000	1:2000	1:2000	1:2000	1:500	1:2000	1:2000
P3.15	2-1-P15	WRAIR 8B5-5-G9	IgG2a	1.50,000	1.50,000	1.50,000	1.50,000	1.50,000	1.50,000	1:5000	1.50,000	1.50,000
P3.21	14-1-P21	WRAIR 6B11-F2-C11	IgG2a	1.50,000	1.50,000	1.50,000	1.50,000	1.50,000	1.50,000	1:5000	1.50,000	1.50,000
**B**												
P1.1		NVI MN14C2.3	IgG2a	1:500	1:500	1:500	1:500	1:500	1:500	1:100	1:500	1:500
P1.2		NVI MN16C13F4	IgG2a	1:500	1:500	1:500	1:500	1:500	1:500	1:100	1:500	1:500
P1.3	12-1-P1.3	WRAIR 5G8B2F9	IgG2a	1:1,000	1:1,000	1:1,000	1:1,000	1:1,000	1:1,000	1:50	1:1,000	1:1,000
P1.4		NVI MN20B9.34	IgG2a	1:2,500	1:2,500	1:2,500	1:2,500	1:2,500	1:2,500	1:100	1:2,500	1:2,500
P1.5		NVI MN22A9.19	IgG2a	1:2,500	1:2,500	1:2,500	1:2,500	1:2,500	1:2,500	1:100	1:2,500	1:2,500
P1.6		NVI MN19D6.13	IgG3	1:500	1:500	1:500	1:500	1:500	1:500	1:100	1:500	1:500
P1.7		NVI MN14C11.6	IgG2a	1:1,000	1:1,000	1:1,000	1:1,000	1:1,000	1:1,000	1:100	1:1,000	1:1,000
P1.9		NVI MN5A10F	IgG2a	1:500	1:500	1:500	1:500	1:500	1:500	1:100	1:500	1:500
P1.9		IAL5F81A4		1:500	1:500	1:500	1:500	1:500	1:500	1:100	1:500	1:500
P1.10		NVI MN20F4.17	IgG2b	1:1,000	1:1,000	1:1,000	1:1,000	1:1,000	1:1,000	1:500	1:1,000	1:500
P1.12		NVI MN21A7.10	IgG3	1:500	1:500	1:500	1:500	1:500	1:500	1:100	1:500	1:500
P1.13		NVI MN25H10.75	IgG2a	1:500	1:500	1:500	1:500	1:500	1:500	1:100	1:500	1:500
P1.14		NVI MN21G3.17	IgG3	1:1,000	1:1,000	1:1,000	1:1,000	1:1,000	1:1,000	1:100	1:1,000	1:1,000
P1.15		NVI MN3C5C	IgG3	1:500	1:500	1:500	1:500	1:500	1:500	1:500	1:500	1:500
P1.16		NVI MN5C11G	IgG2b	1:500	1:500	1:500	1:500	1:500	1:500	1:500	1:500	1:500
P1.19	2-1-P1.19	WRAIR 7A2-11	IgG3	1.50,000	1.50,000	1.50,000	1.50,000	1.50,000	1.50,000	1:500	1.50,000	1.50,000

WRAIR = Walter Reed Army Institute of Research (USA).

NVI = Netherlands Vaccine Institute (previously the RIVM).

Adolfo Lutz Institute (IAL). The Mabs were prepared in Immunology Section by EN De Gaspari.

RT = Room Temperature (30 days).

dilution(a) (Mab) freezer -20°C.

dilution(b) (Mab) eluted of dried filter paper.

Mabs = monoclonal antibodies.

A = Serotyes.

B = Subtypes

**Table 3:** Dot- blot ELISA of class 5 using Monoclonal antibodies for N. meningitidis.

		**1 week**			**4 weeks**	**1 year**	**4oC**	**13 years**	**4 weeks**	**RT**
Mabs		dilution(a)	dilution(b)			dilution(a)	dilution(b)		dilution(a)	dilution(b)
P5.3	WRAIR3BH-C7	1.50,000	1.50,000	1:10,000	1:10,000	1:10,000	1:10,000	1:1000	1:10,000	1:10,000
P5.4	WRAIR1BG11	1.50,000	1.50,000	1:2,500	1:2,500	1:2,500	1:2,500	1:2,500	1:2,500	1:2,500
P5.5	WRAIR3DH-F5G9	1.50,000	1.50,000	1.50,000	1.50,000	1.50,000	1.50,000	1:2,500	1:2,500	1:2,500
P5.8	IALC14F10Br2	1.50,000	1.50,000	1.50,000	1.50,000	1.50,000	1.50,000	1:2,500	1.50,000	1.50,000
P5.9	7F11B5Br3	1.50,000	1.50,000	1.50,000	1.50,000	1.50,000	1.50,000	1:2,500	1.50,000	1.50,000

WRAIR = Walter Reed Army Institute of Research (USA).

NVI = Netherlands Vaccine Institute (previously the RIVM).

Adolfo Lutz Institute (IAL). The Mabs were prepared in Immunology Section by EN De Gaspari.

RT = Room Temperature (30 days).

dilution(a) (Mab) freezer -20°C.

dilution(b) (Mab) eluted of dried filter paper.

Mabs = monoclonal antibodies

**Table 4:** Dot- blot ELISA of immunotypes using Monoclonal antibodies for *N. meningitidis*.

**Specificity**	**Mabs**			**1 week**		**4 weeks**		**1 year**		**13 years**	**4 weeks**	**RT**
			isotypes	dilution(a)	dilution(b)	dilution(a)	dilution(b)	dilution(a)	dilution(b)	dilution(b)	dilution(a)	dilution(b)
L3,7,9	9-2-L379	WRAIR 4BE12C10	IgG2a	1:100,000	1:100,000	1:100,000	1:100,000	1:100,000	1:100,000	1:10,000	1:100,000	1:100,000
L8	6E7-10	WRAIR6E710	IgM	1:100,000	1:100,000	1:100,000	1:100,000	1:100,000	1:100,000	1:10,000	1:100,000	1:100,000
L8	1C31B8	IAL	IgM	1.50,000	1.50,000	1.50,000	1.50,000	1.50,000	1.50,000	1:10,000	1.50,000	1.50,000
L1	1B81C3	IAL	IgM	1:10,000	1:10,000	1:10,000	1:10,000	1:10,000	1:10,000	1:10,000	1:10,000	1:10,000
L1	3G3-1-8C	WRAIR17-1-L1	n/d	1.50,000	1.50,000	1.50,000	1:10,000	1:10,000	1:10,000	1:10,000	1:10,000	1:10,000

WRAIR = Walter Reed Army Institute of Research (USA).

NVI = Netherlands Vaccine Institute (previously the RIVM).

Adolfo Lutz Institute (IAL). The Mabs were prepared in Immunology Section by EN De Gaspari.

RT = Room Temperature (30 days).

dilution(a) (Mab) freezer -20°C.

dilution(b) (Mab) eluted of dried filter paper.

Mabs = monoclonal antibodies

### Monoclonal spot test

For the spot test, monoclonal antibodies for *N meningitides*, in the form of ascites or culture supernatant (10 μL), were applied on a 0.5–1 cm diameter area of Whatman grade 903 paper. The Mabs were dried for 18 h at RT before laboratory testing.

### Collection, transport and storage

All paper strips with Mabs prepared were put into individual self-sealing (ziplock) polythene bags with a few grains of silica to keep out moisture. The Mabs were decanted into polypropylene vials and stored in a freezer at -20°C. The samples were collected once a month by the research staff. Half of these bags were stored at room temperature (25–30°C) and the rest at -20°C until analysis.

### Storage of dried MAb spots on filter paper

Specimens were stored under two conditions. The first was in a freezer at -20°C, and the second was a non-air-conditioned room in the laboratory at a temperature ranging from 15 to 25°C. All Mabs were tested at 4 weeks. Mabs from ascites or culture supernatant, and dried Mab spots on filter paper were determined in parallel. Tables 1, 2, 3, 4 show the comparison between filter paper Mabs of different isotypes of IgG and IgM analyzed by dot-blot ELISA of filter paper eluate at different times.

### Reconstitution of sample from filter papers

The disks containing 10 μL of ascites or culture supernatant were punched out of the filter paper [9]. The paper disks were soaked in 200 μL of phosphate-buffered saline (PBS) supplemented with 0.2% gelatin for 18 h at 4°C on a platform shaker (New Brunswick Scientific Model 2R) at 75 oscillations per min. Eluates contained the equivalent of a 1/20 dilution. The following day the supernatant was stirred, collected and analyzed, together with the corresponding Mab pairs stored frozen at -20°C. Each Mab dilution was previously determined using dot-blot ELISA for *N.meningitidis *reference strain [10].

### Dot-blot ELISA

The method used for serotyping and serosubtyping was essentially that of Abdillahi and Poolman [6]. For preparation of samples (whole cells) for dot-blot, cells were suspended in PBS, pH 7.7, containing 0.02% sodium azide. The cells were heat-inactivated at 56°C for 30 min and the absorbance of the suspension was adjusted to 0.1 at 650 nm using a spectrophometer (model Spectronic 88). The bacterial cell suspension was stored at 4°C. For dot-blot ELISA, 1 μL of meningococcal cell suspension was spotted on 0.22-μm nitrocellulose strips (BioRad). After drying, the strips were incubated for 1 h in blotting buffer containing 5% gelatin (Sigma Chemical Co., St. Louis, Mo.), in PBS. Mabs (Table 1, 2, 3, 4) were pipetted directly into the blocking buffer, diluted as previously determined and incubated continuously overnight at 4°C. The strips were washed separately with PBS six times. Antibody binding was detected after 2 h incubation with a 1:2500 IgG or 1:5000 IgM of rabbit anti-mouse immunoglobulin conjugated to peroxidase (Kirkegaard & Perry Laboratories, Inc., Gaithersburg, MD.). The reaction was visualized by adding a freshly prepared solution containing H_2_O_2 _(30%) and as substrate 0.04% of 3-amino-9-ethyl carbazole(AEC) (Pierce).

## Results

**Figure 1:**
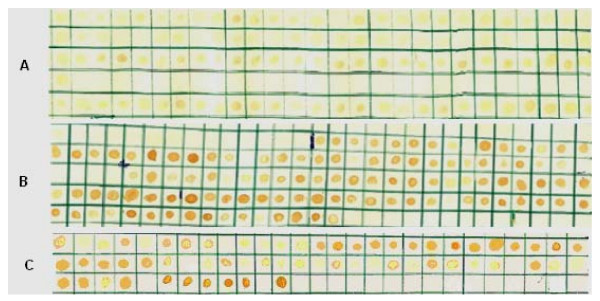
**Dot- blot ELISA using monoclonal antibodies for serogroups A, B and C eluted from filter paper stored for one year at -20°C.** Dot-blot ELISA results of analysis of 323 case strains of *N meningitidis *. The strains was dotted (1 μL) as whole cells suspension.A) Reactivity of the Mab WRAIR 2D7B5B5B2 at dilution 1:25,000(serogroup A); B) Reactivity of the Mab WRAIR 5C1-3H7 at dilution 1:50,000 (serogroup B) and C) Reactivity of the Mab WRAIR 7H94 at dilution 1:25,000 (serogroup C).

The results of the serological tests performed with ascites or culture supernatant samples and dried Mab spots on paper kept at -20°C or at room temperature are presented in Tables 1, 2, 3, 4. The titers were mostly equal and the differences did not alter the final results when the dried Mab spots were stored at -20°C or at room temperature for a period of one year. After 13 years storage, the titers of IgG and IgM antibody isotypes did not interfere with the specificity (Figure 1) of the antibodies when analyzed by dot-blot ELISA. However, none of the differences resulted in a change in serotyping with epidemic strains when we used Mabs of serogroups A, B or C eluted after one year at -20°C (Figure 1). We also included in assays control culture supernatant lacking antibodies to *N.meningitidis *to look for false positives. In all cases where Mab samples were antibody-negative, the corresponding Mab samples dried on dot-Blot ELISA paper tested negative after one week, 4 weeks, 1 year or 13 years of storage at -20°C. Most of the Mabs used in the present investigation are included at the website of, (University of Oxford, UK) and were produced by Dr.W.D Zollinger and Dr. J.T Poolman. Comparison between filter paper Mab and freezer Mab can be seen in Tables 1, 2, 3, 4. The application of these MAbs for serogroup identification of meningococci was demonstrated by their abilities to correctly identify 323 clinical isolates in Brazil by dot blot-ELISA (Figure 1). Serogrouping of *N.meningitidis *(or meningococci) is important because the disease caused by some serogroups can be prevented by active immunization [24].

We also analyzed a series of murine monoclonal antibodies to serotypes and subtypes of *N meningitidis *that were specific for outer membrane proteins of classes 1, 2 or 3. In our laboratory, we prepared and used a Mab to P1.9 strains and others [9,10,25] (Table 2).

The use of monoclonal antibodies for serotyping of meningococci is feasible and easy to perform, and it appears to have significant advantages over the use of polyclonal typing sera [7,26-29]. The analyses using several class 5 Mabs to opacity proteins (Opa), a family of antigenically variable outer membrane proteins of *N. meningitidis *[30], also did not show variability according to dot-blot ELISA after 1 year as dried spots on filter paper (Table 3)

Monoclonal antibodies (Mabs) have largely replaced rabbit antisera as the LOS typing reagent, but a set of Mabs that recognizes most of the important LOS antigens is not available, and many of the available Mabs lack a complete structural definition of their cognate epitopes [31-33]. The pathogenic Neisseria, *N.gonorrhoeae *and *N.meningitidis*, possess an outer membrane protein(OMP), designated H.8, with a conserved Mab binding epitope [34], which was also shown be stable in our studies using a new Mab that was produced earlier [35]. Other cross reactive Mabs also remained stable for one year on dry filter paper at -20°C (data not shown).

It is known that the titer of the Mabs used in dot-blot ELISA for serotyping depends on several factors, mainly on the quality of the anti-IgG or IgM employed as well as the choice of substrate. In our study, we used AEC, which shows a high sensitivity. Before choosing the titer of the Mabs to be used, we carried out a study with different dilutions using reference strains [10] (data not shown).

Another factor examined in this study was the type of filter paper utilized. In an earlier study, we used Whatman 1 MM and observed that the type of filter paper used did not result in an appreciable change in Mab elution [9]. However, in the present study, we used Whatman grade 903 paper.

## Discussion

Typing of *N. meningitidis *includes the determination of groups, serotypes, and subtypes by either whole-cell ELISA or dot-blot ELISA using Mabs. The serotypes, subtypes, LPS, class 5 and cross-reactive specificity of Mabs are defined by their reactivity with whole cells or native outer membrane proteins of the strains by immunoblot analysis.

Despite its limitations, the Mab-based serotyping and serosubtyping method is attractive because it is easy to perform, is relatively inexpensive, and does not require sophisticated equipment. This method is ideal for use in developing countries. The preparation and characterization of additional MAbs and further standardization of the method will increase the utility of this method.

In an ideal world, strains could be characterized using a wide selection of the available typing techniques to create the fullest picture possible of the infective organisms. In practice, this is rarely necessary, and time and financial constraints limit the choices available; therefore, one needs to consider which procedure is the most suitable for a specific situation [36,37].

Thus this method, which is a simple alternative to complicated conventional methods of collection, transport and storage of biological specimens, may be applied to monoclonal antibodies for a host of pathogens. The method is being used routinely in this laboratory for diagnostic and screening purposes with regard to epidemic strains.

Public health specialists are especially interested in the early detection of outbreaks. Filter paper has been used to collect blood for public health purposes for more than 40 years. The paper is made from high-purity cotton linters and is manufactured to give accurate and reproducible absorption of blood specimens according to specifications of the National Committee for Clinical Laboratory Standards (NCCLS) [38]. In this study we used Mabs filter papers with different specifivities that were stored in a freezer for a long period of time. This may have an advantage because we have a large quantity of IgG or IgM antibody for one epitope. In addition, it simulates the reality of using filter papers, usually under conditions where electrical power is not available. We paid special attention to keeping the filter papers dry at room temperature and found no significant reduction in antibody titer after one month, in order to simulate the transport. The detection of IgG or IgM Mabs eluted from filter papers is a useful tool for epidemiologists [39-43].

In summary, MAb samples dried on filter paper and stored for periods of time at -20°C, and later eluted, give results concordant with those of corresponding to Mab samples tested by dot-ELISA. Here, we describe the use of a large number of Mabs of different isotypes and different specificities for antigens of *N. meningitids*. Our laboratory has been working for more than 10 years on the development of new monoclonal antibodies against this pathogen [9,10,25,35,44] and also with others pathogens [45-50]. When we need to send our monoclonals to confirm their utility, this becomes an efficient approach. However, here most of the Mabs used for serotyping this pathogen were used in the characterization of a new monoclonal antibody.

This study has created a mechanism for future validation of filter paper handling and storage of monoclonal antibodies.

## Conclusion

The determination of the most appropriate screening methods and subtype analysis depends on the immediate requirements of an investigation. A panel of well-characterized subtype-specific MAbs proposed for use in screening a large number of isolates during an outbreak should be selected to identify the majority of strains. This method of epidemiologic screening has proven effective in assessing outbreaks during the last 17 years.

The agreement between filter paper eluates and Mabs of IgG or IgM isotypes was substantially high. The agreement was higher with frozen Mabs and the corresponding filter paper Mab eluates. Therefore, in outbreak situations as well as in routine surveillance of this disease, the results of serogroup determination may lead to decisions on the public health intervention measures to be taken. Serogroup determination may also help in understanding the changing epidemiology of meningococcal disease. Murine hybridoma monoclonal antibodies (MAbs) were produced against the capsular antigens of serogroups B, C, Y, W135, 29E, Y and Z meningococci. Each serogroup-specific MAb reacted with the extracted capsular polysaccharide from its homologous serogroup only and did not react with capsules from the three other serogroups.

## Competing interests

The author(s) declare that they have no competing interests.

## Authors' contributions

All authors contributed equally to the research. ASF, EFTB, LMCCC, APO, AMSC, AYY, MSFM are Master's students under the supervision of EDG who had used or are using the monoclonal antibodies from our laboratory. MCGS was a research technician that managed the growth and control of the *N.meningitidis *strains used in this study, DLF was a fellow of PAP/IAL/CCD/SES-SP (2005–2006), EDG was responsible for design of all the study.

## Pre-publication history

The pre-publication history for this paper can be accessed here:


